# Cascade genetic screening in families with hereditary transthyretin amyloidosis: diagnostic and prognostic impact

**DOI:** 10.1093/eurheartj/ehag222

**Published:** 2026-04-07

**Authors:** Francesco Cappelli, Carlo Fumagalli, Marco Luigetti, Roberta Mussinelli, Simone Longhi, Pietro Guaraldi, Alberto Aimo, Alessia Argirò, Alessandro Barilaro, Elena Biagini, Giulia Biagioni, Marco Ceccanti, Alberto Cipriani, Cristina Chimenti, Laura De Michieli, Gianluca Di Bella, Michele Emdin, Francesca Graziani, Massimo Imazio, Giuseppe Limongelli, Carla Lofiego, Francesco Musca, Paolo Ossola, Mario Nuvolone, Stefano Perlini, Maurizio Pieroni, Aldostefano Porcari, Beatrice Musumeci, Giuseppe Palmiero, Federico Perfetto, Irene Ruotolo, Massimo Russo, Giacomo Tini, Giuseppe Vergaro, Fabio Vagnarelli, Federica Verrillo, Maria Ausilia Sciarrone, Alessandro Salvalaggio, Mattia Zampieri, Carlotta Mazzoni, Gianfranco Sinagra, Giovanni Palladini, Marco Merlo, Laura Obici

**Affiliations:** Tuscan Regional Amyloidosis Centre, Careggi University Hospital, Florence, Italy; Department of Experimental and Clinical Medicine, University of Florence, Florence, Italy; Tuscan Regional Amyloidosis Centre, Careggi University Hospital, Florence, Italy; Department of Advanced Medical and Surgical Sciences, University of Campania—Luigi Vanvitelli, Naples, Italy; Fondazione Policlinico A. Gemelli IRCCS, UOC Neurologia, Largo Agostino Gemelli 8, Roma 00168, Italy; Dipartimento di Neuroscienze, Università Cattolica del Sacro Cuore, Largo Agostino Gemelli 8, Rome 00168, Italy; Department of Molecular Medicine, University of Pavia, Pavia, Italy; Cardiology Unit, Cardiac Thoracic and Vascular Department, IRCCS Azienda Ospedaliero-Universitaria di Bologna, Bologna, Italy; European Reference Network for Rare, Low Prevalence and Complex Diseases of the Heart (ERN GUARD-Heart), Bologna, Italy; Biomedical and Neuromotor Sciences—DIBINEM, University of Bologna, Bologna, Italy; Cardiology Division, Fondazione Toscana Gabriele Monasterio, Pisa, Italy; Tuscan Regional Amyloidosis Centre, Careggi University Hospital, Florence, Italy; Tuscan Regional Amyloidosis Centre, Careggi University Hospital, Florence, Italy; Department of Neuromusculoskeletal and Sense Organs, Careggi University Hospital, Florence, Italy; Cardiology Unit, Cardiac Thoracic and Vascular Department, IRCCS Azienda Ospedaliero-Universitaria di Bologna, Bologna, Italy; European Reference Network for Rare, Low Prevalence and Complex Diseases of the Heart (ERN GUARD-Heart), Bologna, Italy; Tuscan Regional Amyloidosis Centre, Careggi University Hospital, Florence, Italy; Department of Human Neurosciences, Rare Neuromuscular Diseases Centre, Sapienza University of Rome, Rome, Italy; Department of Cardio-Thoraco-Vascular Sciences and Public Health, University of Padua, Padua, Italy; Department of Clinical Internal, Anesthesiological and Cardiovascular Sciences, Sapienza University of Rome, Rome, Italy; Department of Cardio-Thoraco-Vascular Sciences and Public Health, University of Padua, Padua, Italy; Rare Cardiac Disease Center, Cardiology Unit, University of Messina, Messina, Italy; Cardiology Division, Fondazione Toscana Gabriele Monasterio, Pisa, Italy; Institute of Life Sciences, Scuola Superiore Sant’Anna, Pisa, Italy; Department of Cardiovascular Sciences, Fondazione Policlinico Universitario A. Gemelli IRCCS, Rome, Italy; Cardiothoracic Department, University Hospital Santa Maria della Misericordia, Udine, Italy; Inherited and Rare Cardiovascular Disease, Department of Translational Medical Sciences, University of Campania ‘Luigi Vanvitelli’, Naples, Italy; Department of Cardiology, Lancisi Cardiovascular Center, Marche University Hospital, Ancona, Italy; Division of Cardiology, ‘De Gasperis Cardio Center’, Niguarda Hospital, Milan, Italy; Division of Cardiology, ‘De Gasperis Cardio Center’, Niguarda Hospital, Milan, Italy; Department of Molecular Medicine, University of Pavia, Pavia, Italy; Department of Molecular Medicine, University of Pavia, Pavia, Italy; Department of Experimental and Clinical Medicine, University of Florence, Florence, Italy; Center for Diagnosis and Treatment of Cardiomyopathies, Cardiovascular Department, Azienda Sanitaria Universitaria Giuliano-Isontina, University of Trieste, Trieste, Italy; National Amyloidosis Centre, Division of Medicine, University College London, Royal Free Hospital, London, UK; Department of Clinical and Molecular Medicine, Sapienza University of Rome, Rome, Italy; Inherited and Rare Cardiovascular Disease, Department of Translational Medical Sciences, University of Campania ‘Luigi Vanvitelli’, Naples, Italy; Tuscan Regional Amyloidosis Centre, Careggi University Hospital, Florence, Italy; Department of Experimental and Clinical Medicine, University of Florence, Florence, Italy; Cardiology Unit, Cardiac Thoracic and Vascular Department, IRCCS Azienda Ospedaliero-Universitaria di Bologna, Bologna, Italy; European Reference Network for Rare, Low Prevalence and Complex Diseases of the Heart (ERN GUARD-Heart), Bologna, Italy; Department of Clinical and Experimental Medicine, University of Messina, Messina, Italy; Department of Clinical and Molecular Medicine, Sapienza University of Rome, Rome, Italy; Cardiology Division, Fondazione Toscana Gabriele Monasterio, Pisa, Italy; Department of Cardiology, Lancisi Cardiovascular Center, Marche University Hospital, Ancona, Italy; Inherited and Rare Cardiovascular Disease, Department of Translational Medical Sciences, University of Campania ‘Luigi Vanvitelli’, Naples, Italy; Department of Neurosciences, Catholic University of the Sacred Heart, Rome, Italy; Department of Neuroscience (DNS) and Padova Neuroscience Center (PNC), University of Padua, Padua, Italy; Tuscan Regional Amyloidosis Centre, Careggi University Hospital, Florence, Italy; Tuscan Regional Amyloidosis Centre, Careggi University Hospital, Florence, Italy; Center for Diagnosis and Treatment of Cardiomyopathies, Cardiovascular Department, Azienda Sanitaria Universitaria Giuliano-Isontina, University of Trieste, Trieste, Italy; Department of Molecular Medicine, University of Pavia, Pavia, Italy; Center for Diagnosis and Treatment of Cardiomyopathies, Cardiovascular Department, Azienda Sanitaria Universitaria Giuliano-Isontina, University of Trieste, Trieste, Italy; Department of Molecular Medicine, University of Pavia, Pavia, Italy

**Keywords:** ATTRv, Genetic screening, Therapy, Outcome

## Abstract

**Background and Aims:**

Hereditary transthyretin amyloidosis (ATTRv) is an autosomal dominant disease with variable penetrance. Cascade genetic screening may enable earlier diagnosis and intervention, but its prognostic impact remains unclear.

**Methods:**

This study retrospectively analysed 967 individuals from 431 families between 2004 and 2024 across 15 Italian referral centres. Participants were categorized as ATTRv index cases, symptomatic carriers (genotype-positive/phenotype-positive [G+/P+]), or asymptomatic carriers (genotype-positive/phenotype-negative [G+/P−]). Clinical characteristics, disease conversion, and survival were evaluated.

**Results:**

Following identification of 398 index cases, genetic screening of 1243 relatives identified 569 carriers (461 G+/P−, 108 G+/P+). Among the 461 G+/P−, over a median follow-up of 5.3 [1.7–9.8] years, 77 (16.7%) patients developed a clinical diagnosis of ATTRv: Glu89Gln (42.2%, 95% confidence interval [CI] 28.8–56.9), Phe64Leu (24.7%, 95% CI 16.1–35.8), Val30Met (13.1%, 95% CI 7.4–22.1), Ile68Leu (7.3%, 95% CI 4.1–12.8), and Val122Ile (5.1%, 95% CI 1.3–18.3), other variants 22.9% (95% CI 14.5–34.1). Notably, 11/62 (17.7%) carriers converted >10 years earlier than the predicted age of disease onset. G + P+ patients had better survival than index (hazard ratio [HR] 0.43, 95% CI 0.24–0.79), and mixed phenotype showed worse outcomes than cardiac presentations. Disease-modifying therapy was independently associated with lower mortality (HR 0.11, 95% CI 0.01–0.17).

**Conclusions:**

Cascade genetic screening facilitated earlier diagnosis and was associated with improved survival, likely related to identification at an earlier stage of disease and timely treatment initiation. Variant-specific follow-up is essential, as some carriers convert earlier than predicted. Systematic, genotype-informed surveillance in ATTRv is key to optimize outcomes.

## Structured Graphical Abstract

**Structured Graphical Abstract:**
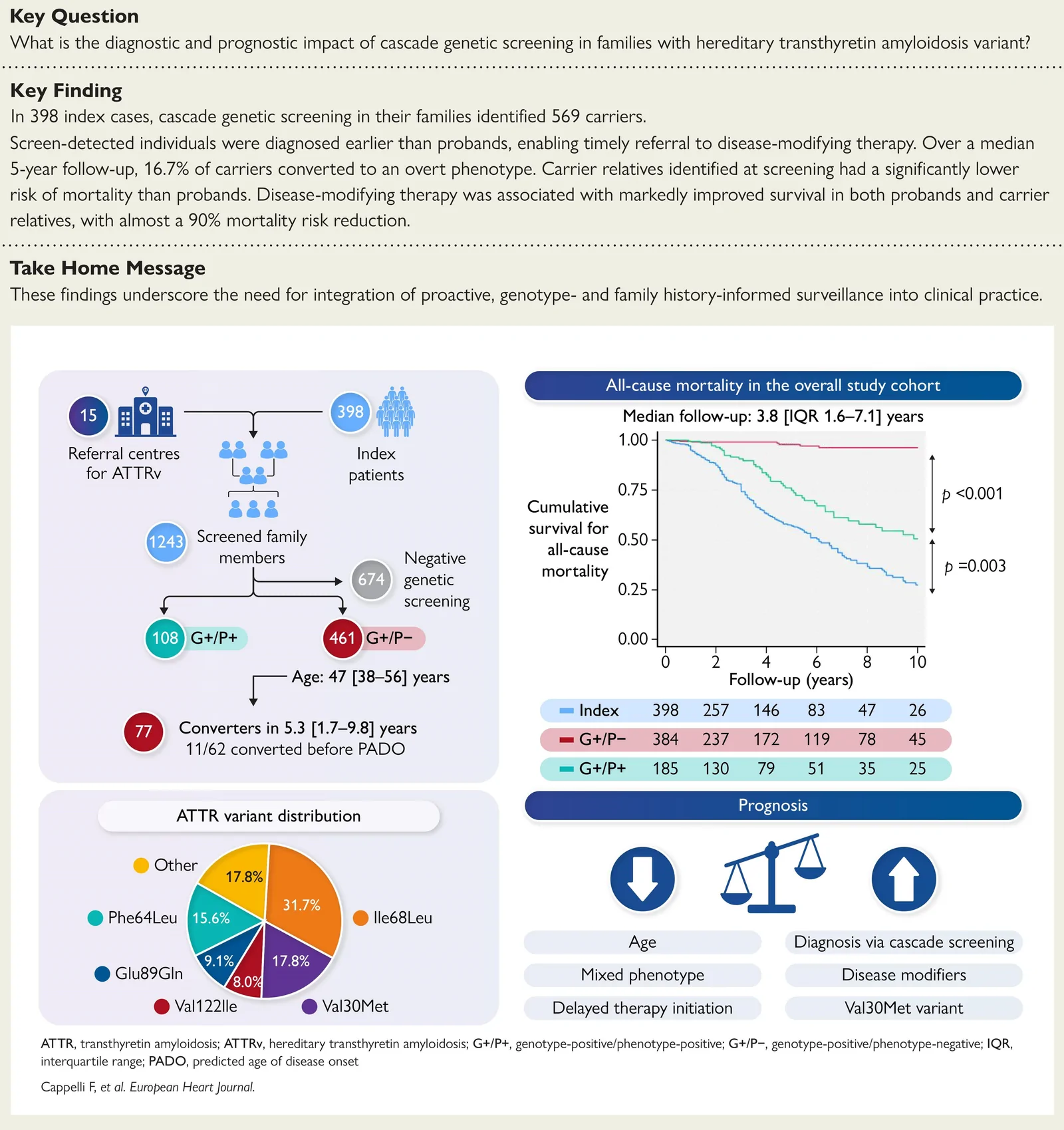
Cascade genetic screening in hereditary transthyretin amyloidosis across 15 referral centres identified 569 carriers (genotype-positive/phenotype-negative, genotype-positive/phenotype-positive), enabling earlier diagnosis, timely initiation of disease-modifying therapy, and improved survival through timely treatment initiation and identification at an earlier stage of disease. Prognostic benefit was evident even after adjustment, with notable genotype-specific differences in conversion before predicted age of disease onset.

**See the editorial comment for this article ‘Cascade genetic screening in hereditary transthyretin amyloidosis: when early diagnosis becomes survival’, by N. Mora-Ayestarán *et al*., https://doi.org/10.1093/eurheartj/ehag063.**

## Introduction

Hereditary transthyretin amyloidosis (ATTRv) is an autosomal dominant disease caused by genetic defects of the transthyretin gene (*TTR*) causing misfolding of the transthyretin (TTR) protein, resulting in amyloid fibril deposition, primarily (but not exclusively) in the heart and peripheral nerves.^1^ Disease expression varies by mutation, ranging from prevalent sensorimotor polyneuropathy with autonomic dysfunction to predominant cardiomyopathy, and often consists of a mixed phenotype.^2^

Cascade genetic testing has been adopted in other hereditary cardiovascular conditions, demonstrating beneficial effects for at-risk family members allowing early and tailored management.^3,4^ With the recent introduction of disease-specific drugs, it has been proposed that in ATTRv, early identification through family screening can improve outcomes by facilitating timely interventions.^5^ Indeed, detecting presymptomatic or mildly symptomatic individuals could potentially provide the opportunity to initiate disease-modifying treatments before irreversible organ damage occurs.

The penetrance of ATTRv is not fully understood,^6–8^ and while screening is recommended approximately 10 years before the predicted age of disease onset (PADO) according to mutation and family history,^9,10^ the rate and age of conversion may vary widely by gender, ethnicity, and other unknown factors.^6^ As the Italian universal healthcare system provides comprehensive coverage for presymptomatic genetic testing, monitoring, and treatment, Italy represents an ideal setting to test the clinical impact of cascade genetic screening in ATTRv, also considering the presence of several referral centres, and the prevalence of different pathogenic genetic variants across the country. In addition, cascade genetic screening is generally well accepted by patients and their relatives, as the Italian laws ensure strict regulation of genetic information and privacy.^11^

With the present study, we aimed to describe the diagnostic and prognostic impact of cascade genetic testing for ATTRv, focusing on prevalence of the disease among screened family members, variant conversion rates, and on long-term outcomes.

## Methods

### Study centres and patient population

A total of 15 referral centres for ATTRv, expert in the diagnosis and management of the disease, participated in this retrospective study. Five were located in Northern Italy (Milan, Padova, Pavia, Trieste, Udine), eight in Central Italy (Ancona, Bologna, Florence, Pisa, Rome Sant’Andrea Hospital, Rome Gemelli Hospital, and Rome Umberto I Hospital), and two in Southern Italy (Naples and Messina). Diagnosis of ATTRv and staging were performed according to the evolving clinical practice and specific guidelines^1,12–14^ (see Supplementary data online, *Table S1*). From 2004 to 2024, families receiving a diagnosis of ATTRv were offered genetic counselling and presymptomatic testing in at-risk relatives according to each centre’s local practice.

The study was approved by the local Ethics Committees, and all participants gave written informed consent for their clinical data to be used for research purposes in accordance with the Declaration of Helsinki.

### Baseline clinical evaluation in index cases

Data on baseline demographic characteristics, clinical evaluation, and instrumental tests were retrospectively collected. Information regarding carriers’ conversion to overt phenotype and date of referral to therapy were also recorded.

### Cascade genetic screening, monitoring of mutation carriers, and definition of disease onset

Starting from the diagnosis of ATTRv in index cases, all subjects identified during family screening carrying a TTR pathogenic variant were included. Genetic testing was first offered to first-degree relatives (siblings and offspring) and then extended also to second-degree relatives. TTRv detection was performed on extracted DNA according to centre practice. Cascade genetic screening has always been routinely proposed across all participating centres. According to each local centre practice, ATTRv mutation carriers were then evaluated by a multidisciplinary team to identify or exclude signs or symptoms of overt ATTR disease. Subjects with clinical, instrumental, or biopsy-proven signs of ATTRv disease at the time of genetic screening (baseline) were identified as genotype-positive/phenotype-positive (G+/P+), whereas those with a negative multidisciplinary baseline evaluation were considered asymptomatic carriers (namely genotype-positive/phenotype-negative; G+/P−). Patients were followed per centre practice with a multidisciplinary evaluation every 6, 12, or 24 months according to PADO and centre practice. In particular, index patients and G+/P+ individuals diagnosed at baseline were reevaluated every 6 months, as per standard practice for symptomatic patients. G+/P− patients were monitored according to their proximity to PADO based on the proband family member (index) age at disease onset: those within 10 years of PADO were generally seen annually, while those more than 10 years from PADO were followed every 2 years.^1^ In addition, PADO was also determined following the time from the average age at diagnosis of the index and the G+/P+ relative according to the family pedigree.^15^

### Phenotype definition

For mutation carriers that developed an overt ATTRv phenotype (defined as the first clinical diagnosis of ATTRv—neuropathy and/or cardiomyopathy—meeting consensus criteria at follow-up), age at conversion, approximate date of phenotype onset and the diagnostic tests indicating conversion were recorded. Incidence was determined based on the time passed from genetic testing to phenotype definition. Standard assessment of patients with positive genetic testing is summarized in Supplementary data online, *Table S1* and was equal for index patients and carriers. All individuals underwent comprehensive cardiovascular and neurologic screening. Signs and symptoms suggesting potential disease included: dyspnoea, peripheral swelling, presence of atrial or ventricular brady- or tachy-arrhythmias, presence or onset of ventricular hypertrophy or other common cardiovascular red-flags,^1^ autonomic dysfunction (e.g. erectile dysfunction, orthostatic hypotension, syncope), gastrointestinal symptoms, unexplained weight loss, bilateral carpal tunnel syndrome, lumbar canal stenosis, renal impairment, ocular involvement, and/or family history of polyneuropathy. The polyneuropathy disability score (PND) consists of five stages: (i) sensory disturbances with preserved walking capacity, (ii) impaired walking capacity with no need for walking aids, (iiia) need for a stick or a crutch for walking, (iiib) two sticks or crutches required for walking, and (iv) wheelchair or bed bound.^1,16^

Cardiac magnetic resonance was performed according to current guidelines and standard clinical practice.^1^ Criteria for diagnosis of ATTRv amyloidosis were as follows: (i) bone scintigraphy (^99m^Tc-DPD, ^99m^Tc-PYP or ^99m^Tc-HMDP) showing cardiac uptake grade II–III according to Perugini score in the absence of a monoclonal gammopathy, (ii) Perugini grade I cardiac uptake with a confirmatory cardiac or extracardiac biopsy, (iii) biopsy-proven cardiac TTR amyloidosis performed for patients with cardiac imaging suggestive of cardiomyopathy without scintigraphy, and (iv) symptoms compatible with ATTRv neuropathy with at least one confirmatory instrumental test and/or a positive (skin) biopsy.^14^

All diagnostic assessments were interpreted locally at each participating centre.

Electrocardiographic abnormalities included rhythm disturbances (atrial fibrillation, conduction blocks (first-degree, second-degree atrioventricular blocks), QRS interval prolongation beyond 120 ms and pseudo-necrosis pattern.

### Study objectives

The primary objectives of the present analysis were (i) to evaluate the prevalence of G+/P+ and carrier individuals among those relatives who performed genetic testing, (ii) to determine variant-specific phenotypic conversion rates and identify tests allowing early detection of conversion, and (iii) to assess the prognostic impact of cascade genetic screening exploring long-term outcome across index cases and patients diagnosed through genetic screening.

### Statistical analysis

Continuous variables are expressed as median and interquartile (IQR) and were compared with nonparametric tests, while categorical variables are expressed as counts and percentages and were compared with χ^2^ or Fisher’s exact test, if the predicted count was <5.

Patients were divided into groups according to cascade family screening as follows: index (defined as the first individual within a family diagnosed with ATTRv, whose diagnosis initiates cascade genetic screening to identify other at-risk family members), G+/P+ (defined as individuals who carry a pathogenic *TTR* mutation and exhibit clinical signs or symptoms of ATTRv), and G+/P− (i.e. *carriers*; defined as individuals who carry a pathogenic *TTR* mutation but do not yet show clinical manifestations of the disease at the time of screening).

For each carrier developing overt disease, date of the first diagnostic test was recorded and was used to determine rates of conversion.

Survival analysis was performed with the Kaplan–Meier method. For cumulative incidence of all-cause mortality, rates were expressed as events per 100 patient-years, where ‘patient-year’ represents the sum of individual follow-up times expressed in years. For index, G+/P+, and carriers without conversion, follow-up started on the day that the genetic test was performed. For carriers manifesting phenotype conversion, the date of follow-up started with the date of conversion by study protocol. In addition to determining incidence rate, a Cox regression analysis was performed to determine the potential factors associated with conversion to over phenotype. Sensitivity analyses were performed by censoring patients who were receiving disease-modifying therapies or enrolled in clinical trials at the start date of these interventions. A time-dependent Cox regression analysis was performed to determine factors associated with all-cause mortality among patients with an overt clinical phenotype (variable selection: backward selection with *P* < .10 as threshold). Disease-modifying therapy was analysed as a time-dependent covariate (stsplit), with exposure switching at the date of treatment initiation; potential time-varying effects were modelled using the tvc() option in Stata. Models did not include variables that would be only collected in the presence of specific phenotypes (i.e. New York Heart Association [NYHA] class or PND scores). Follow-up ended in March 2024. Statistical analysis was performed with IBM SPSS 29.0 (Armonk, NY, USA) and STATA (v19.0).

## Results

### Prevalence of G+/P+ and carriers and clinical characteristics

**Figure 1:**
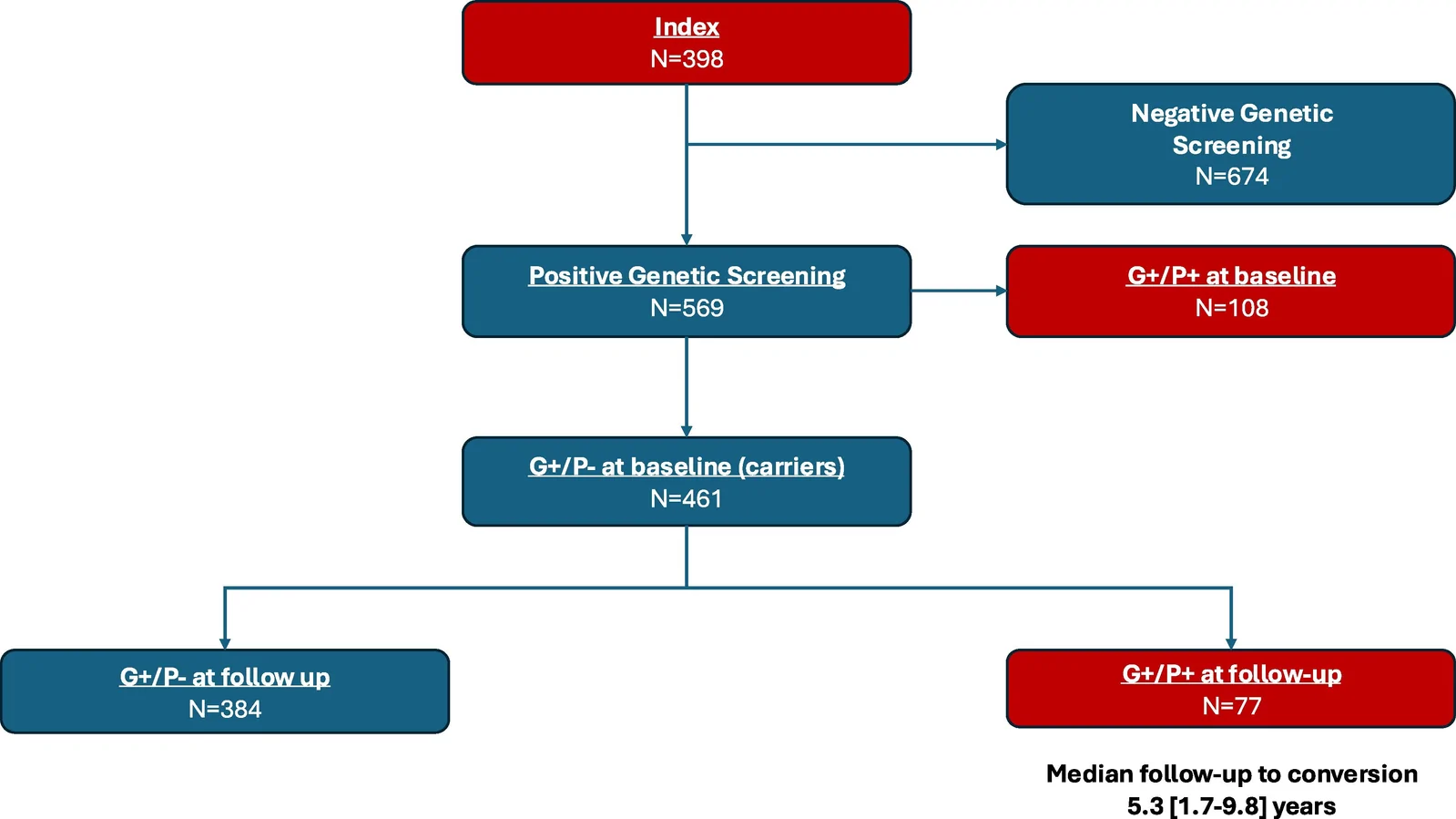
Flowchart of patients enrolled in the study. The flowchart described the derivation of the study cohort, from the identification of the first index patients, to cascade genetic testing and final identification of genotype-positive/phenotype-positive (G+/P+), carriers (genotype-positive/phenotype-negative [G+/P−]) and converted carriers (at follow-up)

In our cohort, 398 index cases were first identified and diagnosed with ATTRv. Following this, 1243 relatives underwent cascade genetic screening, of whom 674 tested negative and 569 (45.8%) were found to carry a pathogenic TTR variant. Of these, 461 were asymptomatic (G+/P−) at the time of genetic testing (98 [21.3%] siblings and 363 [78.7%] offspring of index cases). A total of 137/569 (24.1%) variant carriers were second-degree relatives. Of note, 108 individuals were found to be already affected at the time of genetic testing (G+/P+) (71 [65.7%] siblings and 37 [34.3%] offspring of index cases) (*Figure 1*). Baseline clinical characteristics according to the type of referral and genetic screening are summarized in *Table 1* with a further stratification related to prescription of disease-modifying drugs (DMDs) in Supplementary data online, *Table S2*: of note, carriers and G+/P+ patients increased over time from before 2012 to 2019, as well as the number of individuals receiving DMDs, likely reflecting improved surveillance and diagnostic awareness.

**Table 1:** Baseline clinical characteristics of ATTRv patients enrolled in the study

	Index *n* = 398	Cascade screening		*P*-value Overall comparisons	*P*-value G+/P− vs. G+/P+
		G+/P− *n* = 461	G+/P+ *n* = 108		
Age at time of genetic test (years), median [IQR]	70 [62–76]	47 [38–56]	57 [49–73]	<.001	<.001
Men, *n* (%)	306 (76.9)	210 (45.6)	57 (52.8)	<.001	.001
Ethnicity, *n* (%)					
Caucasian	395 (99.2)	458 (99.4)	108 (100)		
Afro-Caribbean	3 (0.8)	2 (0.4)	0	.561	.889
Other	0	1 (0.2)	0		
Family relationship					
Sibling	-	98 (21.3)	71 (65.7)	<.001	
Offspring	-	363 (78.7)	37 (34.3)		
Variants, *n* (%)				.001	.034
Ile68Leu	126 (31.7)	150 (32.5)	18 (16.7)		
Glu89Gln	36 (9.1)	45 (9.8)	28 (25.9)		
Val122Ile	32 (8.0)	39 (8.5)	3 (2.8)		
Phe64Leu	62 (15.6)	73 (15.8)	14 (13.0)		
Val30Met	71 (17.8)	84 (18.2)	25 (23.1)		
Other	71 (17.8)	70 (15.2)	20 (18.5)		
Matrilineal inheritance, *n* (%) (*n* = 518)	-	148 (38.5)	18 (34.6)	.584	
Phenotype, *n* (%) (*n* = 957)				<.001	
ATTRv-CA	145 (37.4)	-	26 (24.3)		
ATTRv-PN	67 (17.3)	-	39 (36.4)		
Mixed	175 (45.1)	-	42 (39.3)		
Negative	-	461 (100.0)	-		
NYHA class, *n* (%)				<.001	
I	126 (31.7)	-	65 (60.2)		
II	187 (47.0)	-	26 (24.1)		
III−IV	59 (14.8)	-	6 (5.6)		
PND score, *n* (%) (*n* = 447)	347		100	.001	
0	115 (33.1)	-	23 (23.0)		
1	107 (30.8)	-	47 (47.0)		
2	80 (23.1)	-	23 (23.0)		
3a	25 (7.2)	-	5 (5.0)		
3b	8 (2.6)	-	0		
4	12 (3.4)	-	2 (2.0)		
eGFR (mL/min/1.73 m^2^), median [IQR] (*n* = 350)	71 [56–91]	94 [89–100]	85 [67–94]	.002	<.001
NT-proBNP (pg/mL), median [IQR] (*n* = 292)	1337 [432–3141]	27 [23–88]	442 [141–1122]	.001	<.001
Atrial fibrillation, *n* (%)	102 (25.6)	7 (1.5)	6 (5.3)	<.001	.011
Pseudo-infarction pattern, *n* (%)	51 (12.8)	4 (0.9)	9 (8.3)	<.001	<.001
Low voltage, *n* (%)	33 (8.3)	6 (1.3)	7 (6.5)	<.001	.005
IV conduction block, *n* (%)	30 (7.5)	2 (0.4)	5 (4.6)	<.001	.030
Type I AV block, *n* (%)	28 (7.0)	1 (0.2)	3 (2.8)	.009	.023
Maximum LVWT (mm), median [IQR] (*n* = 830)	16 [14–19]	10 [9–11]	13 [11–19]	<.001	.001

ATTRv, hereditary transthyretin amyloidosis; AV, atrioventricular; CA, cardiomyopathy; eGFR, estimated glomerular filtration rate; IQR, interquartile range; IV, intraventricular; LVWT, left ventricular wall thickness; NT-proBNP, N-terminal pro-B-type natriuretic peptide; NYHA, New York Heart Association; PN, neuropathic; PND, polyneuropathy disability.

Median age at diagnosis was highest among index cases, compared to their G+/P+ relatives. Male prevalence was highest among index patients, followed by G + P+ and carriers (*Table 1*).

At diagnosis a mixed phenotype was observed in 175 (45.1%) of index cases, higher than G+/P+ where it was present in 42 (39.3%) of G+/P+ individuals (*P* < .001). Neurological symptoms were more prevalent in affected relatives, as seen in PND scores. Renal function, as assessed by estimated glomerular filtration rate (eGFR), was significantly lower in index patients (median 71 mL/min/1.73 m^2^) compared to carriers (94 mL/min/1.73 m^2^) and G+/P+ individuals (85 mL/min/1.73 m^2^) (*P* = .002).

Overall, atrial fibrillation was the most frequent abnormality at ECG, followed by pseudo-infarction pattern, low voltages, intraventricular conduction blocks and type I atrioventricular blocks: these had a significantly higher prevalence among index patients, followed by G+/P+ and carriers.

### Characteristics of carriers who developed an overt disease

**Figure 2:**
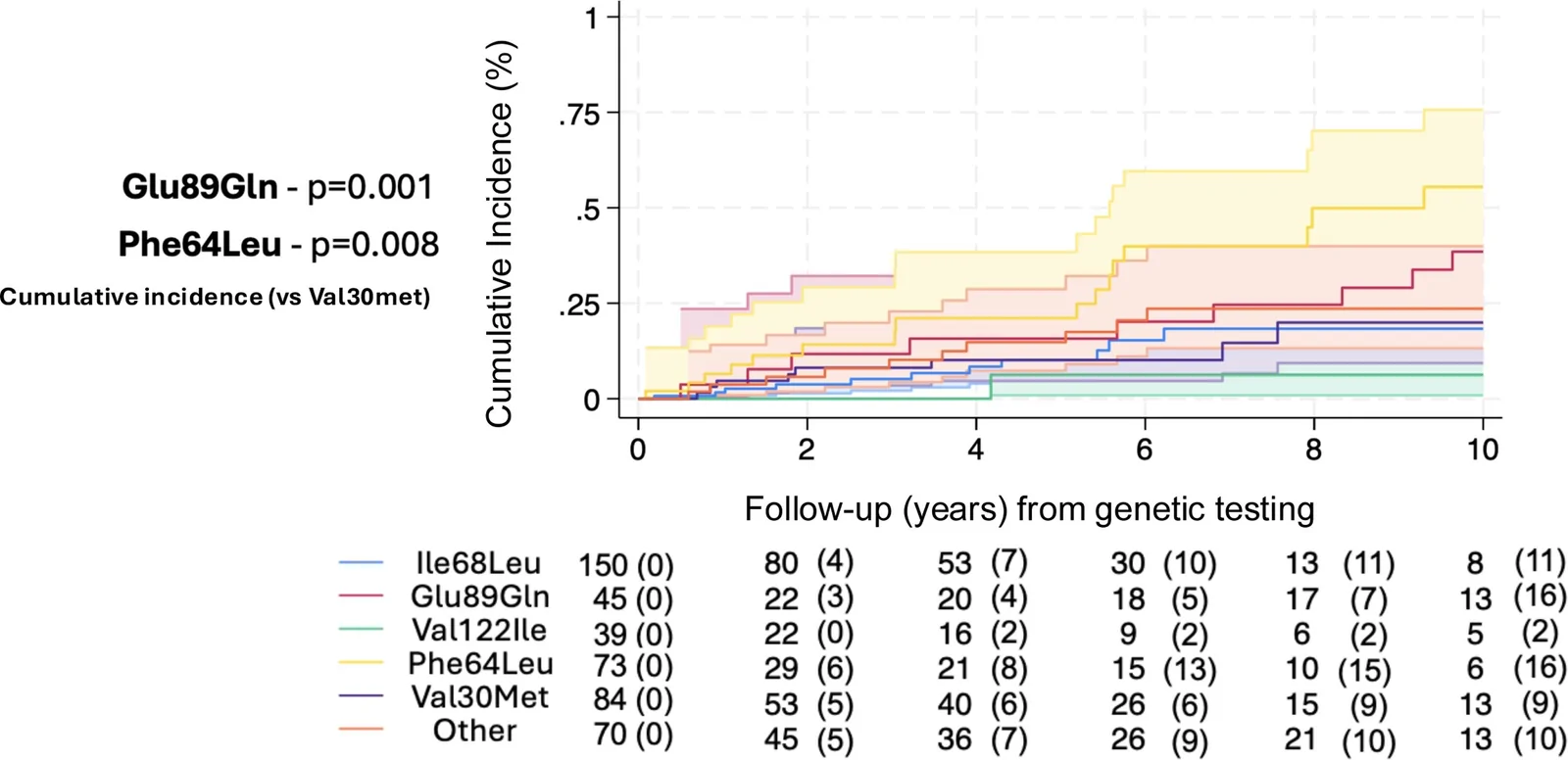
Incidence of conversion of overt clinical phenotype among carriers at follow-up. Cumulative incidence curves with 95% confidence intervals (shaded areas) and numbers at risk are shown, highlighting the significantly higher conversion rates for Glu89Gln and Phe64Leu variants. Numbers in brackets represent cumulative converted cases within the time frame presented. Follow-up time started at genetic testing. Pairwise comparison (vs Val30Met): Ile68Leu: *P* = .641, Glu89Gln: *P* = .001, Phe64Leu: *P* = .008; Val122Ile: *P* = .404, Other: *P* = .623

Among the 461 carriers (G+/P−), over a median follow-up time of 5.3 [1.7–9.8] years, 77 (16.7%) patients, including 33 (42.9%) siblings and 44 (57.1%) offspring of index cases, developed a TTR-related form of disease. The rate of conversion varied significantly by genetic variant and age group (*Figure 2*, *Table 2* and Supplementary data online, *Figure S2*): among carriers, the highest conversion rate was seen in Glu89Gln (42.2%, 95% CI]28.8–56.9), followed by Phe64Leu (24.7%, 95% CI 16.1–35.8), Val30Met (13.1%, 95% CI 7.4–22.1), Ile68Leu (7.3%, 95% CI 4.1–12.8), and Val122Ile (5.1%, 95% CI 1.3–18.3). Other variants accounted for 22.9% (95% CI 14.5–34.1). A significantly higher risk of conversion of Glu89Gln was also confirmed at Cox regression analysis after adjusting for gender and age (see Supplementary data online, *Table S3*). The median age of conversion for all carriers was 59.6 [IQR 50.0–69.2] years, with conversion occurring earlier in carriers of Glu89Gln (median age 49.1 [IQR 47.7–52.1] years) compared to other variants.

**Table 2:** Age distribution, conversion rates, and clinical characteristics of genetic mutation carriers leading to overt phenotypes

	Overall	Ile68Leu	Glu89Gln	Val122Ile	Phe64Leu	Val30Met	Other	*P*-value
Age of index cases at baseline (years), median [IQR]	N = 398	N = 126	N = 36	N = 32	N = 62	N = 71	N = 71	
	70 [62–76]	75 [69–79]*	54 [50–60]*	74 [70–78]	69 [64–74]*	70 [65–73]	61 [47–71]	<.001
Age of G+/P+ at baseline (years), median [IQR]	N = 108	N = 18	N = 28	N = 3	N = 14	N = 25	N = 20	
	57 [49–73]	73 [56–78]*	54 [49–55]*	76 [69–82]*	75 [65–77]	60 [42–67]*	48 [41–54]*	<.001
Age at baseline of carrier’s that do not convert to overt phenotype (years), median [IQR]	N = 384	N = 139	N = 26	N = 37	N = 55	N = 73	N = 54	
	47 [38–55]	50 [43–58]*	34 [28–40]*	47 [40–52]	46 [37–52]	45 [38–52]	39 [32–49]*	<.001
Percentage of carriers that convert to overt phenotype	Overall 77/461 (16.7)	Ile68Leu 11/150 (7.3)	Glu89Gln 19/45 (42.2)	Val122IIe 2/39 (5.1)	Phe64Leu 18/73 (24.7)	Val30Met 11/84 (13.1)	Other 16/70 (22.9)	
Age of carriers at baseline who convert (years), median [IQR]	54.5 [39.0–65.8]	63.2 [53.7–68.3]*	38.7 [36.0–48.4]*	57, 78	57.5 [53.0–67.0]	63.0 [52.5–68.1]	38.8 [25.4–58.3]*	<.001
Age at conversion (years), median [IQR]	59.6 [50.0–69.2]	66.5 [58.8–71.7]	49.1 [47.7–52.1]	68, 82	62.8 [55.8–74.2]	66.8 [61.5–72.3]	42.8 [31.3–51.6]	<.001
Siblings, *n* = 33	67.9 [59.2–72.4]	72.5 [70.1–73.7]	52.2 [51.3–58.2]	57, 78	71.9 [63.1–74.8]	71.9 [65.2–74.8]	60.8 [57.7–70.3]	<.001
Offspring, *n* = 44	52.1 [46.3–60.4]	59.3 [57.7–62.2]	49.3 [47.0–51.1]	-	59.4 [54.0–63.5]	60.4 [55.6–66.3]	38.8 [31.5–44.8]	<.001
Time to conversion	5.3 [1.7–9.8]	3.2 [1.3–4.8]	9.8 [5.6–15.0]	11.0, 4.1	5.2 [1.3–7.8]	3.4 [1.3–8.8]	4.3 [2.2–14.4]	<.001
Diagnosed >10 years before the index relative age at symptoms onset, *n* (%)	11/62	5/11	3/16	0/2	2/15	1/10	0/9	.098
Diagnosed >10 years before the index relative age at onset and G + P+ family member diagnosis, *n* (%)	17/65	6/11	1/16	0/2	3/15	3/10	4/12	.065
Men, *n* (%)	40 (51.9)	8 (72.7)	11 (57.9)	1 (50.0)	8 (44.4)	8 (72.7)	4 (25.0)	.104
Matrilinear inheritance, *n* (%)	24/52	3/10	6/14	-	4/10	1/4	10/14	.233
Phenotype at conversion, *n* (%)								.029
ATTRv-CA	29 (37.7)	7 (63.6)	7 (36.8)	2 (100)	2 (11.1)	2 (18.2)	7 (43.8)	
ATTRv-PN	46 (59.7)	3 (27.3)	10 (52.6)	0	16 (88.9)	9 (81.2)	8 (50.0)	
Mixed	4 (5.2)	1 (0.9)	2 (10.5)	0	0	0	1 (6.2)	
First test suggesting conversion, *n* (%) in 75 patients	***N* = 75**	***N* = 10**	***N* = 18**	***N* = 2**	***N* = 18**	***N* = 11**	***N* = 16**	
Echocardiography	24 (32.0)	7 (70.0)	8 (44.4)	1 (50.0)	4 (22.2)	0	4 (25.0)	.142
Cardiac magnetic resonance	5 (6.7)	2 (20.0)	1 (5.6)	0	1 (5.5)	0	1 (6.3)	
Neurologic testing	46 (61.3)	2 (20.0)	10 (55.5)	0	16 (88.8)	7 (63.4)	11 (68.8)	
Test confirming diagnosis^a^								
Scintigraphy, *n* (%)	28 (37.3)	7 (70.0)	9 (50.0)	2 (100)	2 (11.1)	2 (18.2)	6 (37.5)	
Neurologic testing, *n* (%)	42 (56.0)	2 (20.0)	8 (44.4)	0	15 (83.3)	7 (63.4)	10 (62.5)	
Biopsies, *n* (%)	32	-	8	1	7	6	10	
Fat pad, *n* (%)	21 (65.6)	-	8 (100.0)	1 (100.0)	3 (42.9)	2 (33.3)	7 (70.0)	.057
Skin or other, *n* (%)	11 (34.4)	-	0	0	4 (57.1)	4 (66.6)	3 (30.0)	
Heart, *n* (%)	1 (3.1)	-	1 (12.5)	-	-	-	-	
Neurologic involvement, n/N								
Small fibres	22/34	3/5	6/8	0	6/9	3/5	4/6	.802
Large fibres	37/69	2/7	12/18	0	9/18	5/10	9/14	.267
Neuroautonomic	5/69	0	1/18	0	4/18	0	0	.106
Motor symptoms	17/69	1/7	6/18	0	4/18	3/10	3/14	.892
Sensitivity symptoms	25/69	3/7	6/18	0	6/18	5/10	5/14	.704
Gastro-intestinal symptoms	9/69	0	3/18	0	2/18	0	4/14	.297

ATTRv, hereditary transthyretin amyloidosis; CA, cardiomyopathy; G+/P+, genotype-positive/phenotype-positive; IQR, interquartile range; PN, neuropathic; PND, polyneuropathy disability.

^*^Significant *post hoc* comparisons for continuous variables.

^a^A total of 27 patients performed more than one diagnostic test.

Remarkably, among carriers who converted to overt disease, the proportion of male (51.9%) was lower than that observed in index patients and closely resembled that of G+/P+ individuals diagnosed at baseline (*Table 2*).

When intra-familial age at onset was analysed to determine how many carriers converted beyond the 10-year window, a total of 11/62 carriers developed signs and symptoms before the 10 years from proband’s age (see Supplementary data online, *Figure S1A*). These included: Ile68Leu (*N* = 5/11–45.5%), Glu89Gln (*N* = 3/16–18.8%), Phe64Leu (*N* = 2/15–13.3%), and Val30Met (*N* = 1/10–10.0%). Similar results were obtained when carriers were analysed according to age at diagnosis by family pedigree, with 17 patients developing ATTRv disease prior to 10 years of PADO (see Supplementary data online, *Figure S1B*). We also determined the prevalence of converted carriers only in relatives within the 10 years of the PADO and results were clinically similar: Glu89Gln (40.1%, 95% CI 27.5–55.6), followed by Phe64Leu (21.4%, 95% CI 13.3–32.5), Val30Met (9.8%, 95% CI 5.0–18.6), Ile68Leu (3.4%, 95% CI 1.4–8.1), and Val122Ile (5.1%, 95% CI 1.3–18.3). Other variants accounted for 18.2% (95% CI 10.6–29.4).

Moreover, clinical conversion was frequently represented by evidence of neurological involvement, particularly small fibre neuropathy (22/34) and sensitivity symptoms (25/69). Accordingly, the test confirming clinical conversion was predominantly neurologic (61.3%). This may reflect both the tendency of patients to overlook or tolerate early neurological symptoms and the high prevalence of mixed phenotypes associated with the TTR variants observed in our cohort.

### Long-term survival and outcomes

After disease diagnosis, patients were followed for a median of 3.8 [IQR 1.6–7.1] years (index 3.4 [1.6–5.8] years; G+/P+ 4.2 [1.3–8.5] years; G+/P− 4.6 [2.3–8.6] years). Overall, in 344 of 583 (59.1%) eligible patients (including 398 index cases and 185 [108 at G+/P+ baseline and 77 converters] affected relatives) a DMD (either stabilizer or silencer) was started (Index: *n* = 218, G+/P+: *n* = 126; median time on treatment: 2.9 [1.0–4.2] years and 3.1 [1.0–4.4] years for index and G+/P+, respectively; *P* = .106). In particular, 221/583 (37.9%) received tafamidis (51/221 [23.1%] tafamidis 20 mg), 88/583 (15.1%) patisiran, 21/583 (3.6%) diflunisal, and 14/583 (2.4%) inotersen.

**Figure 3:**
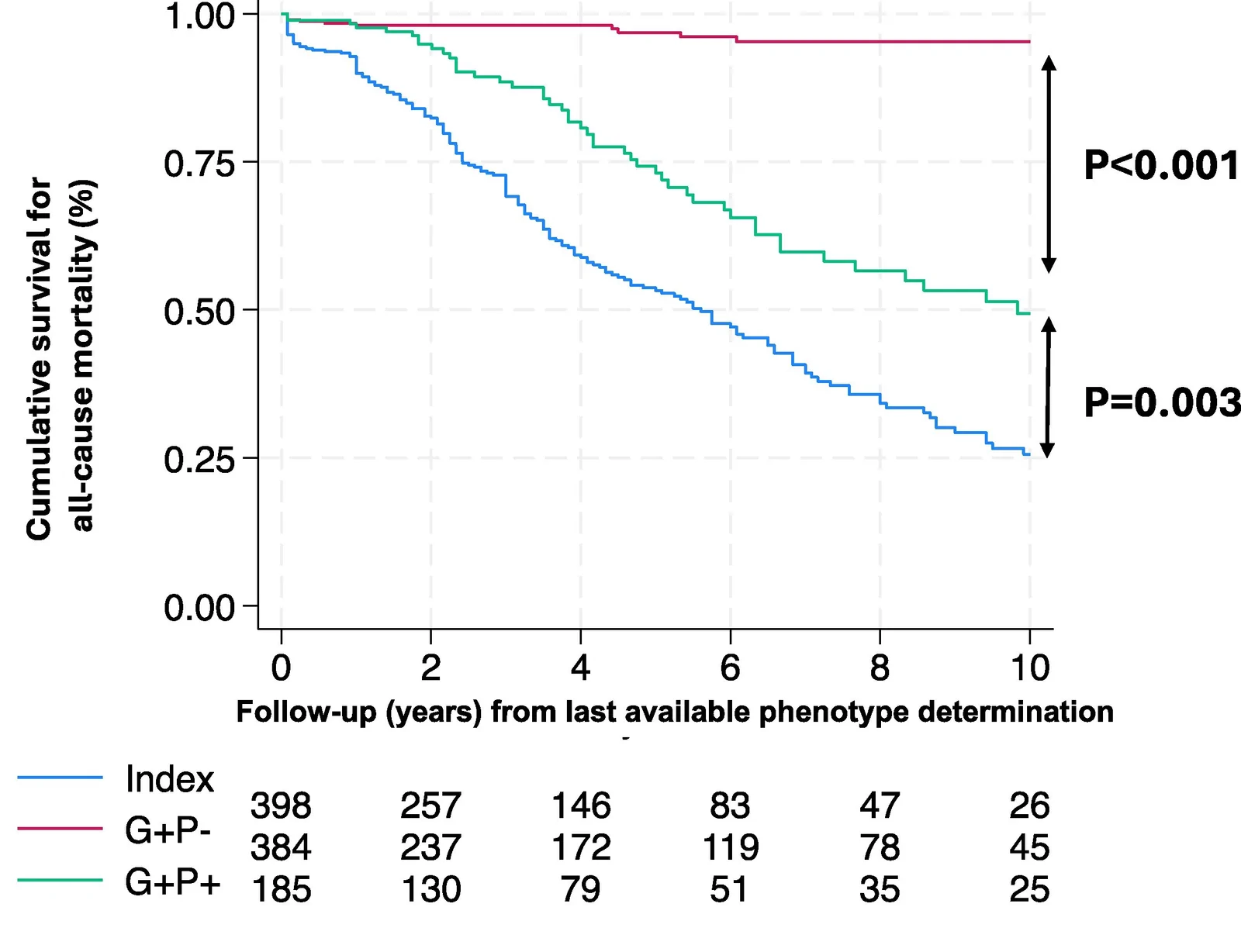
Unadjusted survival analysis of patients enrolled in the study from the last available clinical phenotype. Kaplan–Meier curves show improved survival for G+/P+ patients identified through cascade screening compared to index cases, with follow-up time starting at the last available phenotype determination

During follow-up, 258/967 (26.7%) patients died. Index patients showed a worse survival compared to G + P+, even after adjustment for age, gender, and type of mutation (*Figure 3* and Supplementary data online, *Figure S3*), with a median survival time of 6.0 (95% CI 5.0–6.9) years. Cumulative incidence of overall mortality according to last available phenotype were 10.5 per 100 patient*year (95% CI 9.0–12.3) vs 5.8 per 100 patient*year (95% CI 4.5–7.9) vs 0.5 per 100 patient*year (95% CI 0.3–1.0) for index vs G+/P+ vs G+/P−, respectively (*P* < .001).

**Figure 4:**
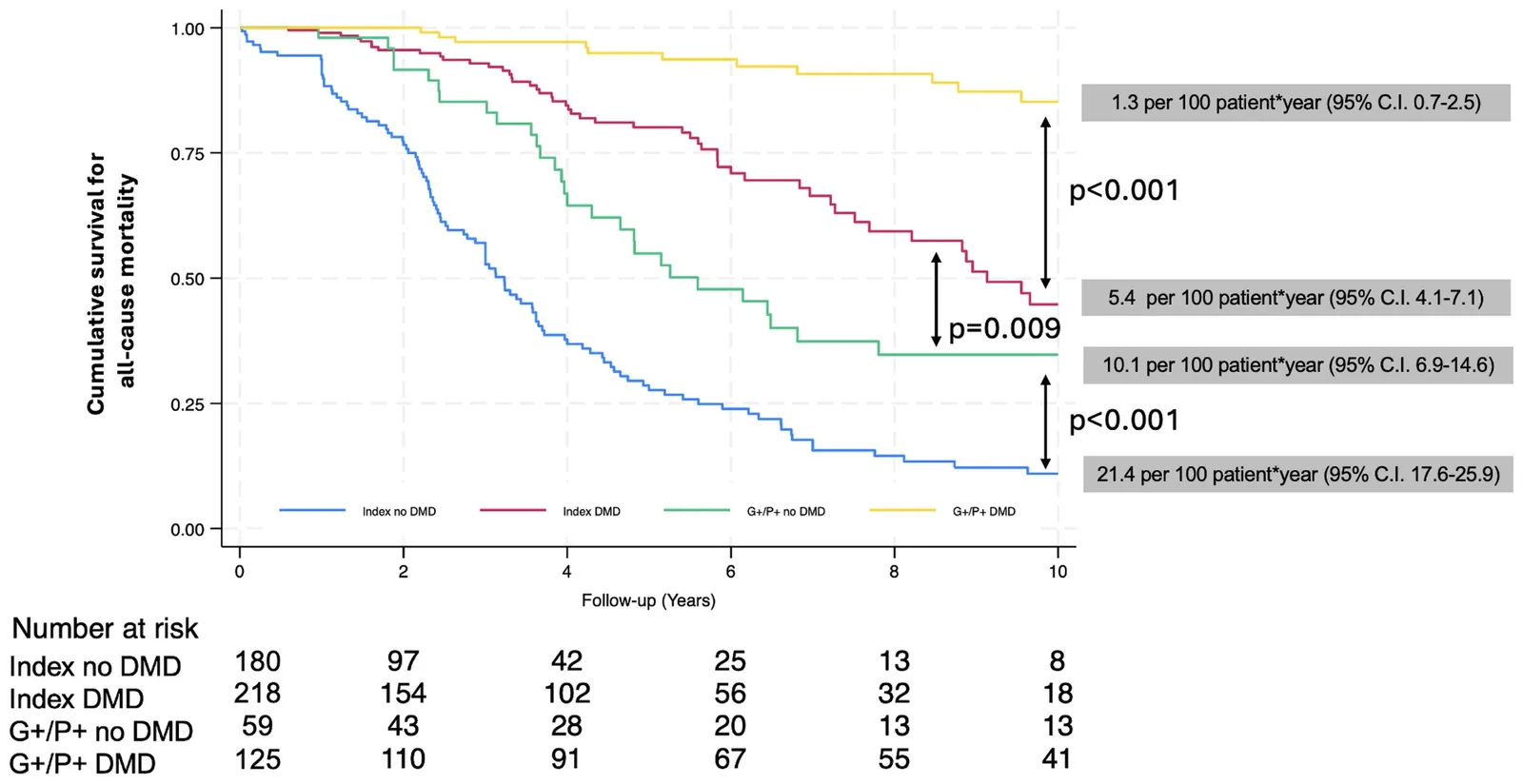
Survival analysis by phenotype and disease-modifying drugs (DMDs). Kaplan–Meier curves show significantly improved survival among patients receiving DMDs, particularly in G+/P+ individuals highlighting the impact of early detection and treatment initiation (all *P* < .01). Follow-up time was defined from the start of DMD therapy for both index and G+/P+ patients (including converted carriers)

Of note, when stratified by treatment, DMDs consistently improved survival both among index and G+/P+ patients (*Figure 4*): while cumulative incidence of all-cause mortality was reduced by >50% in the treated cohort (with index patients referred to DMDs significantly outliving G+/P+ not receiving therapy), G+/P+ patients receiving DMDs also showed a more favorable outcome at follow-up over the entire cohort. Supplementary data online, *Figure S4* presents survival analysis according to phenotype (index vs G+/P+ and converted carriers) receiving DMDs.

At time-dependent Cox multivariable regression analysis (*Table 3*), after adjustment for age, genotype, and disease-modifying therapy, G+/P+ patients diagnosed earlier via screening showed a net survival benefit with a reduced mortality risk (hazard ratio [HR] 0.433, 95% CI 0.238–0.788, *P* = .006). Of note, disease-modifying therapy was associated with reduced mortality risk (HR 0.109, 95% CI 0.005–0.173, *P* < .001), but treatment effect changed over analysis time, indicating a gradual attenuation of the initial protective effect during follow-up (HR 1.112, 95% CI 1.024–1.251, *P* = .014). Furthermore, Val30Met and a pure cardiac phenotype were associated with better survival compared to those with other variants and mixed phenotype.

**Table 3:** Multivariable time-dependent Cox regression analysis to predict all-cause mortality in patients diagnosed with ATTRv

Variable	Hazard ratio	95% CI	*P*-value
Age at genetic diagnosis (per 1-year increase)	1.049	1.034–1.065	<.001
G+/P+ vs Index	0.433	0.238–0.788	.006
Genotype (Val30Met Reference)			
Ile68Leu	1.421	1.004–2.819	.031
Glu89Gln	1.710	1.103–3.012	.038
Val122Ile	1.984	1.141–3.452	.015
Phe64Leu	1.468	0.996–2.618	.051
Other	1.728	1.088–2.997	.022
Phenotype			
Pure cardiac vs mixed	0.737	0.380–0.912	.043
Pure neuropathic vs mixed	1.049	0.676–1.626	.828
Disease modifier	0.109	0.005–0.173	<.001
Time-varying covariate disease modifier	1.112	1.024–1.251	.014

ATTRv, hereditary transthyretin amyloidosis; CI, confidence interval; G+/P+, genotype-positive/phenotype-positive.

## Discussion

In this study we demonstrated that cascade screening in ATTRv allows the early identification of a significant number of additional patients. Early detection of phenotype at the time of genetic diagnosis and early detection of phenotypic conversion during follow-up allowed early initiation of disease-modifying treatments improving the long-term outcome. To the best of our knowledge, this is the first comprehensive investigation of the effectiveness of cascade genetic screening in ATTRv in a nationwide cohort spanning over 20 years.

The main findings of our study can be summarized as follows: (i) cascade genetic screening identified 569 carriers, including 461 G+/P− and 108 G+/P+ family members, (ii) patients identified by screening received a diagnosis earlier than probands, allowing for potentially early referral for disease-modifying therapy, (iii) over a median follow-up of 5 years, 16.7% of G+/P− carriers converted to an overt clinical phenotype and became eligible for disease-modifying therapies: 11/62 converted before the PADO, (iv) disease-modifying treatment was significantly associated with improved clinical outcome in both index patients and G+/P+ relatives, with almost a 90% relative risk reduction in mortality, and (v) early treatment initiation in patients identified by cascade screening, either affected at baseline or developing the phenotype during follow-up, was associated with a more favourable risk profile compared to index patients (*Structured Graphical Abstract*).

Taken together these findings highlight the clinical relevance and reinforce the indication of cascade genetic screening in ATTRv. These findings further support the importance of a structured multidisciplinary care model—including neurology, cardiology, clinical genetics and, when appropriate, rehabilitation and ophthalmology—since neurological manifestations are common in variant carriers and may be under-recognized within cardiology-led pathways.

### Impact of cascade genetic screening on ATTRv epidemiology

The demographic and clinical profiles of patients identified through cascade screening differed significantly from those of index patients. Over time, this practice showed the potential to reshape the epidemiology of ATTR-related disease and supports the need for genotype-, gender-, and age-specific screening approaches. This aligns with recent national data showing that, over the past two decades, improved awareness, advances in diagnostic tools, and wider genetic screening access have accelerated diagnosis, increased prevalence and incidence, and shifted the mutation spectrum towards mixed and cardiac phenotypes.^17^ Moreover, in our cohort, the yield of screening asymptomatic carriers increased in the later years of the study, likely reflecting this growing awareness, simplified diagnostic pathways, and earlier use of noninvasive imaging within structured screening programmes.

The G+/P+ cohort identified at baseline through family screening included both older siblings and younger offspring, reflecting the wide intrafamilial variability in disease expression. While this heterogeneity likely contributed to their intermediate clinical profile across cardiac and neurological assessments, differences in eGFR across groups should be interpreted with caution, as renal function may vary due to age and comorbidities and does not necessarily indicate amyloid-related renal involvement. Notably, the G+/P+ group was on average younger than probands, yet already showed clinical manifestations, and included a higher proportion of women—nearly 50%—a figure that exceeds what is typically reported in the literature.^17,18^ Given the autosomal dominant inheritance pattern of ATTRv, this finding raises the possibility that, still today, many women remain undiagnosed, not because of lower penetrance *per se*, but due to under-diagnosis.^15,19^ Although the underlying reasons remain uncertain, one possible explanation involves the growing prevalence of the Ile68Leu and other predominantly cardiac variants, for which diagnostic thresholds such as septal wall thickness may exhibit important sex-specific differences.^17,20^

In addition to gender-specific considerations, the high proportion of G+/P+ individuals among offspring—accounting for approximately one-third of cases—challenges the common practice of screening strategies based on the predicted age at symptom onset in the proband.^1^ This need is further supported by our finding that among the 16.7% of carriers converting to overt phenotype, a nonnegligible number of carriers developed symptoms more than 10 years earlier than the proband’s age at onset at both methods of PADO determination (determined either according to the age at disease onset in the family proband or according to average at disease diagnosis in the family pedigree). These early converters were clustered among carriers of the Ile68Leu, Glu89Gln, Phe64Leu, and Val30Met variants—which, although uncommon, highlights the substantial variability in age at onset associated with these variants.

Overall, while these findings are consistent with recent data supporting the feasibility and effectiveness of European Society of Cardiology (ESC)-recommended cascade screening protocols to identify specific ATTRv phenotypes,^18^ they also explore and extend the indication to testing and to routine multidisciplinary assessments even in the presence of mild symptoms (especially when specific variant carriers) in order to prioritize early diagnosis and determine eligibility to disease modifiers.

### Impact of cascade genetic screening on treatment and survival

Beyond early diagnosis, at survival analysis, cascade family screening was also associated with a meaningful survival benefit. Over a median follow-up of 3.8 years, overall mortality exceeded 25%, with index patients experiencing higher risk and a significantly higher cumulative incidence of death compared to G+/P+. In time-dependent multivariable Cox regression, patients diagnosed via cascade screening (G+/P+) had a 57% lower risk of mortality relative to probands (HR 0.433, 95% CI 0.238–0.788, *P* = .006), even after adjustment for age, genotype, phenotype, and treatment exposure, indicating that earlier identification through cascade screening is associated with improved prognosis: further confirming this hypothesis, although initiation of disease-modifying therapy was associated with an approximate 90% reduction in mortality risk (HR 0.109, 95% CI 0.005–0.173, *P* < .001), its overall benefit declined with each year of delay, indicating a gradual attenuation of the initial protective effect during follow-up. This trend could really be driven by the probands who started treatment later in their life and have higher event rates despite DMD therapy. Of note, genotype and phenotype were both retained in the multivariable model, as phenotype expression in ATTRv is increasingly recognized to be heterogeneous across variants. Recent multicentre data have shown that *TTR* mutations once considered phenotype-specific (e.g. Val30Met, Ile68Leu, Phe64Leu, Val122Ile) frequently display mixed cardiac-neurological presentations, thereby reducing the risk of major genotype–phenotype collinearity in multivariable analyses.^17^

These observations should also be interpreted in the context of the evolving epidemiology of ATTRv and the historical nature of our cohort. Many index cases were diagnosed before disease-modifying therapies became available or reimbursed, whereas broader cascade screening in more recent years has enabled earlier detection, earlier treatment, and the identification of milder phenotypes—factors that directly influence survival estimates. For these reasons, some degree of residual confounding cannot be excluded. Nevertheless, these data reinforce the clinical value of structured cascade screening as a strategy to improve long-term outcomes in ATTRv, particularly when paired with prompt referral to treatment in the early stages of disease, while also underscoring the importance of considering potential reimbursement and access barriers.

Notably, cascade screening and periodic carrier follow-up can be time- and resource-consuming processes that may result in increased medicalization and anxiety—key modulators of quality of life, especially as it is often more complex given the challenges of communicating age-dependent penetrance.^18^ Recent evidence in related contexts, such as hypertrophic cardiomyopathy, has shown that the yield of genetic diagnosis can be as high as 1-in-4 screened individuals, with similar clinically meaningful long-term consequences.^21^ While these strategies may determine higher upfront costs in the short-term, they are later associated with a favourable cost-effectiveness profile.^22–24^ Importantly, at a time when DMDs are becoming increasingly available, the issue of a proper timing of treatment in mildly symptomatic carriers to prevent further disease burden is not only ethically very relevant but also cannot any longer be postponed. Obviously, ATTRv could benefit from early screening to guide disease-specific therapy and prevent disease progression–related hospitalizations, functional capacity deterioration and death.^15^

Another key finding from our cohort is that 16.7% of G+/P− carriers converted to an overt clinical phenotype over a median follow-up of 5 years. Our findings reinforce the concept that ATTRv is a dynamic continuum, beginning with early TTR amyloid deposition that may remain asymptomatic or subclinical for many years. Progression to overt clinical disease occurs once amyloid burden exceeds a threshold sufficient to impair organ function, a process that can vary greatly according to genotype, age, and other host factors. The observation that a proportion of carriers converted significantly earlier than predicted underscores this heterogeneity, and highlights the importance of genotype-specific, tailored surveillance to enable intervention at the earliest signs of phenotypic transition. Specifically, the rate of conversion, varied significantly by mutation. During a median follow-up of 5.3 [1.7–9.8] years, Glu89Gln carriers showed the highest conversion rate (42.2%), with a much earlier age of conversion compared to other more common pathogenic variants like Ile68Leu and Val122Ile. While we cannot exclude that the duration of follow-up could be in part responsible for this, this heterogeneity suggests a patient-tailored approach once genetic testing is confirmed positive.

Overall, these results align with the recommendations of the International Society of Amyloidosis, the American Heart Association, and the American College of Cardiology, which advocate genetic testing in all patients with ATTR.^25–27^ Our findings extend this message by demonstrating how such testing should be systematically integrated into clinical practice, coupled with genetic counselling, to enable early identification of at-risk relatives—not only in offspring (often carriers) but also in siblings (often presenting with overt phenotype). This approach supports timely access to both disease-modifying therapies and comprehensive supportive care (e.g. pharmacological, rehabilitative, physical, and nutritional interventions). Moreover, our data reinforce the rationale underpinning the ACT-EARLY trial (NCT06563895), emphasizing the strategic importance of early detection and surveillance of asymptomatic carriers to facilitate prompt intervention and improve long-term outcomes.

### Study limitations

The retrospective design over 20 years may introduce bias, particularly in the referral and selection of index patients. However, the multicentre nature of the study including tertiary referral centres specialized in the care of rare diseases with similar screening and monitoring protocols, may have mitigated such potential bias. Follow-up intervals varied across centres (ranging from 6 to 24 months), particularly for asymptomatic carriers, which may have introduced bias in estimating the exact timing of phenotypic conversion, as those on longer surveillance intervals could have had delayed recognition of disease onset. Early subclinical patients may have been missed, leading to potential underestimation of ATTRv disease. Furthermore, given the long study period and the multicenter nature of the cohort, troponins were measured inconsistently and with different assays (including high-sensitivity assays). As such, data on troponins were not collected. Similarly, data on left atrial size, ejection fraction, and global longitudinal strain were not collected. No data on individuals who refused to undergo testing could be retrieved. From a clinical standpoint, data on PND score and NYHA class were recorded only when considered clinically relevant (i.e. when neurological or cardiac involvement was suspected or present); similarly, NAC scores or other potential residual confounding or modulating factors for survival analysis may have not been recorded systematically due to the retrospective nature of the study. Furthermore, only associations between covariates and mortality can be determined. Biopsies, like fat pads, have a reasonable sensitivity for ATTRv, but far from perfect: this may induce underestimation bias.

In addition, the study is focused on a predominantly Italian cohort with regional-specific variant distribution and lower prevalence of some genotypes (like Val122Ile or Thr60Ala). This may limit the generalizability of the findings to other populations, where genetic variants, mutation portfolio, and healthcare infrastructure may differ. On the other hand, this approach highlights the importance of a nationwide network, with shared protocols and collaboration in the setting of a uniform national healthcare system.

Future prospective international collaborative studies with standardized follow-up protocols across diverse populations are needed to validate these findings in different national cohorts, and identify variant-specific surveillance strategies.

## Conclusions

Cascade genetic screening effectively identifies variant carriers with no or milder disease phenotype, favouring early intervention and thus determining a more favourable outcome. Our findings support a mutation-specific follow-up to timely detect early clinical conversion of pathogenic variant carriers. Future prospective international and multicentre studies with shared follow-up protocols are needed to validate these findings in other countries with different epidemiology and genetic background.

## Supplementary Material

ehag222_Supplementary_Data

## Declarations

### Data Availability

Data will be provided upon reasonable request to the corresponding author.
