# Supplementary material for: Cascade genetic screening in families with hereditary transthyretin amyloidosis: diagnostic and prognostic impact
Source: Eur Heart J. 2026 Apr 7;47(32):4462–76. doi: 10.1093/eurheartj/ehag222 (PMC13500165; doi:10.1093/eurheartj/ehag222)
Supplement: Supplementary file 1 [file ehag222_supplementary_data.docx]

**SUPPLEMENTARY MATERIAL**

**Supplementary Table 1**. Standard assessment of patients with positive genetic testing.

**Supplementary Table 2.** Baseline Clinical characteristics of vATTR patients enrolled in the study by genetic screening and referral to disease modifying drugs (DMD).

**Supplementary Table 3.** Multivariable Cox Regression Analysis to predict conversion in carriers of ATTR variants

**Supplementary Figure 1. Distribution of the time difference between the age at carrier conversion and the age at disease onset among ATTRv carriers.** Distribution of the time difference between age at phenotypic conversion in 62 ATTRv carriers and the age at disease onset of their proband family member, highlighting instances of conversion occurring more than 10 years earlier than predicted (PADO, left of the dashed line – Panel A). Panel B presents PADO according to the same principles and based on the age at diagnosis of index patients and family members according to the family pedigree. Each line corresponds to one patient.

**Supplementary Figure 2.** Incidence of Conversion by Genotype and Age bracket.

**Supplementary Figure 3. Model-Adjusted Survival According to Clinical Group**. Model-adjusted survival estimates obtained from a multivariable Cox regression including age, sex, last available clinical phenotype, and TTR variant. Index patients demonstrate the lowest adjusted survival, whereas G+P− carriers show the most favourable prognosis. Follow-up time is anchored at diagnosis for index cases and at phenotype definition for the remaining groups. The accompanying table displays hazard ratios with 95% confidence intervals for all covariates included in the model.

**Supplementary Figure 4. Survival Analysis of patients by phenotype and receiving Disease Modifying Drugs.** Kaplan–Meier curves showing survival of patients treated with disease modifying drugs according to phenotype (index vs G+/P+ at baseline vs converted G+/P-).

**Supplementary Table 1. Standard assessment of patients with positive genetic testing**^1–5^

**Cardiovascular evaluation**

Electrocardiogram and 24h ECG Holter monitoring

Echocardiogram

NTproBNP and troponin*

Cardiac Magnetic Resonance and/or Bone Scintigraphy, every 3 years or earlier under clinical suspicion

**Neurological evaluation**

Neurological examination - General assessment, including Neuropathy Impairment Score (NIS)

Orthostatic blood pressure measurement

Nerve conduction studies (NCSs) - Standard motor and sensory NCSs (e.g. sural, tibial, median, ulnar, dorsal nerves)

Sudoscan and/or electrochemical skin conductance

**Biopsies**

Abdominal fat pad

Cardiac biopsy

Skin biopsy

Nerve biopsy (e.g., sural nerve)

Gastrointestinal Biopsy

*Both standard and high-sensititivity depending by the Center and the time period.

1. Gillmore JD, Maurer MS, Falk RH, et al. Nonbiopsy diagnosis of cardiac transthyretin amyloidosis. *Circulation*. 2016. Published online2016. https://doi.org/10.1161/CIRCULATIONAHA.116.021612.

2. Garcia-Pavia P, Rapezzi C, Adler Y, et al. Diagnosis and treatment of cardiac amyloidosis: a position statement of the ESC Working Group on Myocardial and Pericardial Diseases. *Eur Heart J*. 2021;42:1554–1568.

3. Rauf MU, Hawkins PN, Cappelli F, et al. Tc-99m labelled bone scintigraphy in suspected cardiac amyloidosis. *Eur Heart J*. 2023;44:2187–2198.

4. Adams D, Ando Y, Beirão JM, et al. Expert consensus recommendations to improve diagnosis of ATTR amyloidosis with polyneuropathy. *J Neurol*. 2021;268:2109–2122.

5. Adams D, Sekijima Y, Conceição I, et al. Hereditary transthyretin amyloid neuropathies: advances in pathophysiology, biomarkers, and treatment. *Lancet Neurol*. 2023;22:1061–1074.

| Supplementary Table 2. Baseline Clinical characteristics of vATTR patients enrolled in the study by genetic screening and referral to disease modifying drugs (DMD) | | | | | | |
| --- | --- | --- | --- | --- | --- | --- |
|  | **Index** | | **Cascade Screening** | | | **p** |
|  | **No DMD**  **N=178** | **DMD**  **N=220** | **G+/P-**  **N=461** | **G+/P+**  **No DMD**  **N=48** | **G+/P+**  **DMD**  **N=60** |  |
| **Age at time of genetic test (years), median [IQR]** | 70 [61-77] | 70 [62-75] | 47 [38-56] | 56 [49-67] | 57 [49-75] | <0.001 |
| **Men, N (%)** | 138 (77.5) | 168 (76.4) | 210 (45.6) | 23 (47.9) | 34 (56.7) | <0.001 |
| **Decade at disease diagnosis, N (%)** | **N=155** | **N=217** | **N=448** | **N=46** | **N=60** |  |
| **<2012** | 74 (47.7) | 29 (13.4) | 85 (19.0) | 33 (71.7) | 19 (31.7) | 0.001 |
| **2012-2019** | 57 (36.8) | 104 (47.9) | 213 (47.5) | 7 (15.2) | 19 (31.7) |  |
| **≥2020** | 24 (15.5) | 84 (38.7) | 150 (33.5) | 6 (13.1) | 22 (36.7) |  |
| **Ethnicity, N (%)** |  |  |  |  |  |  |
| **Caucasian** | 176 (98.9) | 219 (99.5) | 458 (99.4) | 48 (100) | 60 (100) |  |
| **Afro-Caribbean** | 2 (1.1) | 1 (0.5) | 2 (0.4) | 0 | 0 | 0.802 |
| **Others** | 0 | 0 | 1 (0.2) | 0 | 0 |  |
| **Variants, N (%)** |  |  |  |  |  |  |
| **Ile68Leu** | 67 (37.6) | 59 (26.8) | 150 (32.5) | 4 (8.3) | 14 (23.3) | 0.001 |
| **Glu89Gln** | 15 (8.4) | 21 (9.5) | 45 (9.8) | 12 (25.0) | 16 (26.7) |  |
| **Val122Ile** | 18 (10.1) | 14 (6.4) | 39 (8.5) | 2 (4.2) | 1 (1.7) |  |
| **Phe64Leu** | 25 (14.0) | 37 (16.8) | 73 (15.8) | 4 (8.3) | 10 (16.7) |  |
| **Val30Met** | 25 (14.0) | 46 (20.9) | 84 (18.2) | 17 (35.4) | 8 (13.3) |  |
| **Other** | 28 (15.7) | 43 (19.5) | 70 (15.2) | 9 (18.8) | 11 (18.3) |  |
| **Matrilineal inheritance, N (%)**  **(available for 518 pts)** | - | - | 148 (38.5) | 6/20 (30.0) | 12/32 (37.5) | 0.584 |
| **Phenotype, N (%) (available for 956 patients)** |  |  |  |  |  |  |
| hATTR-CA | 81 (48.2) | 64 (29.1) | - | 6 (12.8) | 20 (33.3) | <0.001 |
| hATTR-PN | 26 (15.5) | 41 (18.6) | - | 21 (44.6) | 18 (30.0) |  |
| Mixed | 61 (36.3) | 114 (51.8) | - | 20 (42.6) | 22 (36.7) |  |
| Negative | - |  | 461 (100.0) | - |  |  |
| **NYHA class, N (%)** |  |  |  |  |  |  |
| NYHA I | 48 (27.0) | 78 (35.5) | - | 32 (66.7) | 33 (55.0) | <0.001 |
| NYHA II | 71 (39.9) | 116 (52.7) | - | 10 (20.8) | 16 (26.7) |  |
| NYHA III, IV | 41 (23.0) | 18 (8.2) | - | 1 (2.1) | 5 (8.3) | <0.001 |
| **PND score, N (%) (available for 447 patients)** | 136 | 211 |  | 41 | 59 | 0.001 |
| Score 0 | 56 (41.2) | 59 (28.0) | - | 5 (12.2) | 18 (30.5) |  |
| Score 1 | 31 (22.8) | 76 (36.0) | - | 18 (43.9) | 29 (49.2) |  |
| Score 2 | 29 (21.3) | 51 (24.2) | - | 15 (36.6) | 8 (13.6) |  |
| Score 3A | 12 (8.8) | 13 (6.2) | - | 1 (2.4) | 4 (6.8) |  |
| Score 3B | 4 (2.9) | 4 (1.9) | - | 0 | 0 |  |
| Score 4 | 4 (2.9) | 8 (3.8) | - | 2 (2.0) |  |  |
| **eGFR (ml/min/1,73m^2^), median [IQR] (available for 350 patients)** | 66 [49-90] | 76 [60-91] | 94 [89-100] | 78 [59-103] | 86 [75-93] | 0.002 |
| **NT-proBNP (pg/ml), median [IQR] (available for 292 patients)** | 2513 [895-6120] | 1114 [386-2648] | 27 [23-88] | 1047 [236-1813] | 428 [110-895] | 0.001 |
| **Atrial fibrillation, N (%)** | 47 (26.4) | 55 (25.0) | 7 (1.5) | 3 (6.3) | 3 (5.0) | <0.001 |
| **Type I AV block, N (%)** | 10 (5.6) | 18 (8.2) | 1 (0.21) | - | 3 (5.0) | 0.081 |
| **IV conduction blocks, N (%)** | 14 (7.9) | 16 (7.3) | 2 (0.42) | 1 (2.1) | 4 (6.7) | 0.010 |
| **Pseudo-infarction pattern, N (%)** | 26 (14.6) | 25 (11.4) | 4 (0.9) | 5 (10.4) | 4 (6.7) | 0.032 |
| **Low voltage, N (%)** | 19 (10.7) | 14 (6.4) | 6 (1.3) | 3 (6.3) | 4 (6.7) | 0.221 |
| Abbreviations: ATTRv: variant Transthyretin Amyloidosis; CA: Cardiomyopathy; eGFR: estimated Glomerular Filtration Rate; IQR: Interquartile Range; LVWT: Left Ventricular Wall Thickness; NYHA: New York Heart Association; PN: Neuropathic; PND: polyneuropathy disability. | | | | | | |

| **Supplementary Table 3. Multivariable Cox Regression Analysis to predict conversion in carriers of ATTR variants** | | | |
| --- | --- | --- | --- |
| **Variable** | **Hazard Ratio** | **95% Confidence Interval** | **P-value** |
| **Gender (men)** | 1.515 | 0.935-2.454 | 0.091 |
| **Genotype**  **(Val30Met reference)** |  |  |  |
| Ile68Leu | 0.546 | 0.221-1.302 | 0.169 |
| Glu89Gln | 5.032 | 2.242-11.291 | <0.001 |
| Val122Ile | 0.514 | 0.111-2.370 | 0.392 |
| Phe64Leu | 2.708 | 1.235-5.942 | 0.013 |
| Other | 1.637 | 1.161-4.991 | 0.031 |
| **Age at genetic screening (per year increase)** | 1.076 | 1.054-1.098 | <0.001 |

**Supplementary Figure 1**

**Supplementary Figure 2**

**Supplementary Figure 3**

**Supplementary Figure 4**
